# Molecular mechanism of a novel root-end filling material containing zirconium oxide on the osteogenic/odontogenic differentiation of human osteosarcoma MG-63 cells

**DOI:** 10.3389/fbioe.2023.1269246

**Published:** 2023-10-13

**Authors:** Yao-Zhong Chen, Yan Huang, Xiao-Ying Lü

**Affiliations:** ^**1**^ Department of Operative Dentistry and Endodontics, Zhongda Hospital, Medical College, Southeast University, Nanjing, China; ^**2**^ State Key Laboratory of Digital Medical Engineering, School of Biological Science and Medical Engineering, Southeast University, Nanjing, China

**Keywords:** root-end filling material, hydroxyapatite, gene expression profiling microarray, bioinformatics, osteogenic/odontogenic differentiation molecular mechanism

## Abstract

Although the novel root-end filling material containing zirconium oxide (NRFM-Zr) which is hydroxyapatite-based may promote osteoblast differentiation, the molecular mechanism remains unclear. The aim of this study is to investigate it underlying the osteogenic/odontogenic differentiation of human osteosarcoma MG-63 cells induced by NRFM-Zr, compared with calcium silicate-based mineral trioxide aggregate (MTA), and glass ionomer cement (GIC). Firstly, three different types of root filling materials were co-cultured with MG-63 cells, and their cell toxicity, alkaline phosphatase (ALP) activity, and calcium ion concentration were evaluated. Next, gene expression profiling microarray was employed to analyze the impact of the materials on the gene expression profile of MG-63 cells. The results of cell viability revealed that NRFM-Zr group had no significant difference compared to the negative control group. After 5 and 7 days of cultivation, both the NRFM-Zr and MTA groups exhibited significantly higher ALP activity compared to the negative control (*p* < 0.05). Moreover, the NRFM-Zr group had the highest calcium ion concentration, while the GIC group was the lowest (*p* < 0.05). Gene expression profiling microarray analysis identified 2915 (NRFM-Zr), 2254 (MTA) and 392 (GIC) differentially expressed genes, respectively. GO functional and KEGG pathway analysis revealed that differentially expressed genes of NRFM-Zr, MTA and GIC participated in 8, 6 and 0 differentiation-related pathways, respectively. Comparing the molecular mechanisms of osteogenic/odontogenic differentiation induced by hydroxyapatite-based NRFM-Zr and calcium silicate-based MTA, it was found that they shared similarities in their molecular mechanisms of promoting osteogenic differentiation. NRFM-Zr primarily promotes differentiation and inhibits cell apoptosis, thereby enhancing osteogenic/odontogenic differentiation of MG-63 cells. Furthermore, the inducing efficacy of NRFM-Zr was found to be superior to MTA.

## 1 Introduction

When non-surgical retreatment of the root canal fails or cannot be undertaken, apical surgery is generally considered as the preferred treatment option. The main objective of apical surgery is to prevent microorganisms or their by-products from entering the surrounding periapical tissues by filling it with a tight root-end filling material (RFM) following root-end resection (von Arx, 2011). Therefore, the performance of the RFM is one of the key factors that influence the success rate of apical surgery.

Ideal RFM should not only present good biocompatibility, antimicrobial activity, non-toxicity, and radiopacity, but also exhibit osteogenic or odontogenic properties. This is because the RFM should have the capability to form hydroxyapatite, enabling creating a bond between with dentin, thus promoting a physiological seal. Currently, there are numerous root-end filling materials available in clinical practice; however, few of them present osteogenicity and odontogenicity (Koh et al., 1998).

As one of traditional non-bioactive RFMs, glass ionomer cement (GIC) has been widely used in the field of dentistry due to its excellent adhesive properties to dental tissues and the ability to release fluoride ions. However, it lacks bioactivity. Sangsuwan et al. (2015) showed that GIC inhibited alkaline phosphatase (ALP) activity and downregulated the expression of bone morphogenetic protein-2 (BMP-2) and osteopontin (OPN) genes in normal human osteoblasts. Additionally, GIC had been found to inhibit the formation of apatite in simulated body fluid (Kamitakahara et al., 2001).

Unlike traditional non-bioactive RFMs such as GIC and silver amalgam, mineral trioxide aggregate (MTA) is recognized as a bioactive material with properties of hard tissue conductivity and induction (Gandolfi et al., 2010). Torabinejad et al. (1995) showed that out of six root ends filled with MTA, five had complete cementum formation over the filling material, whereas cementum formation was absent on the surface of silver amalgam. Gandolfi et al. (2010) confirmed the formation of a layer of apatite on the surface of ProRoot MTA in a simulated body fluid containing phosphates. As a hydrated calcium-silicate cement material, MTA appears to have become the gold standard for root-end filling materials (von Arx, 2011; Sanz et al., 2021). However, MTA still has challenges such as long setting time, poor handling properties, and so on (Gandolfi et al., 2010; von Arx, 2011).

In recent years, our research group has developed a novel root-end filling material containing zirconium oxide (NRFM-Zr), whose main components are hydroxyapatite, calcium carboxylate, and zirconium dioxide, etc., therefore, it belongs to the hydroxyapatite-based materials (Chen Y. Z. et al., 2013; Chen et al., 2018). NRFM-Zr exhibits favorable physicochemical properties such as good washout resistance, radiopacity, and hydrophilicity, as well as excellent biocompatibility, making it a promising RFM. Moreover, NRFM-Zr is a bioactive material that can enhance ALP activity and mineral deposition in Saos-2 cells, indicating its potential to promote osteoblast differentiation (Chen et al., 2018). However, the molecular mechanism underlying its osteogenic/odontogenic differentiation effect remains unclear.

Gene expression profiling microarray technology enables the efficient and rapid detection and analysis of a large number of genes. When combined with bioinformatics analysis, it allows for a comprehensive study of gene-gene relationships, making it possible to elucidate the molecular mechanisms underlying the different effects of biomaterials on tissues and cells. For instance, gene expression profiling microarray technology has been applied to investigate the molecular mechanisms of osteogenic differentiation in calcium silicate-based materials such as MTA and iRoot BP (Kim et al., 2010; Torun et al., 2016). However, to date, there have been no comparative studies on the osteogenic/odontogenic differentiation mechanisms between calcium silicate-based materials (such as MTA) and hydroxyapatite-based materials (such as NRFM-Zr).

The aim of this study is to first compare the biocompatibility of NRFM-Zr, MTA, and GIC at the cellular level. Subsequently, gene expression profiling microarray technology combined with bioinformatics analysis will be used to investigate the molecular mechanisms underlying the osteogenic/odontogenic differentiation induction of human osteosarcoma MG-63 cells by NRFM-Zr at the molecular level.

## 2 Materials and methods

### 2.1 Materials preparation

The NRFM-Zr powder was composed of 34.4% hydroxyapatite, 34.4% tetracalcium phosphate, 7.2% solid polyacrylic acid, 2.4% solid citric acid, 1.6% sodium citrate, and 20.0% zirconium dioxide (by mass percentage) which was used to improve its radio-opacity (Chen et al., 2018; Mahgoub et al., 2019). NRFM-Zr circular disk samples in two sizes (diameter 5 mm × thickness 2 mm, diameter 20 mm × thickness 1 mm) was prepared as follows: the material powder was mixed with distilled water in a 5:1 mass ratio and then placed in a circular plastic plate which was stored at 37°C and 100% humidity for 1 day (Chen et al., 2018). Subsequently, the samples were sterilized for 12 h in an ethylene oxide sterilizer (Anprolene AN 74ix; Andersen, Haw River, NC, United States). Before conducting biological experiments, the samples were degassed for 7 days to remove residual ethylene oxide and rinsed with sterile phosphate-buffered saline (PBS) (Chen Y. Z. et al., 2013). Additionally, ProRoot MTA (Dentsply Tulsa Dental Specialties, Johnson City, TN, United States of America) and GIC (FX-II, Shofu, Inc., Kyoto, Japan) were used as control materials, which were fabricated according to the manufacturer’s instructions, with powder/liquid mass ratios of 3.3:1 and 2.6:1, respectively (Chen et al., 2018).

### 2.2 Cell viability assay

The MG-63 cells (Institute of Cell Biology, Chinese Academy of Sciences, Shanghai, PRC) were cultured in calcium-containing MEM medium (Gibco Laboratories, Grand Island, United States) containing 10% fetal bovine serum, 0.15% sodium bicarbonate, penicillin (100 U/mL), and streptomycin (100 mg/mL) in a humidified atmosphere containing 5% CO_2_ at 37°C. Logarithmic growth phase MG-63 cells were seeded at a density of 3 × 10^4^ cells per well in a 24-well plate and incubated overnight (Ceci et al., 2015; Margunato, et al., 2015). The culture medium was then removed, and transwells (diameter 6.5 mm, Corning Gilbert Inc., Glendale, AZ, United States) containing test material samples (diameter 5 mm × thickness 2 mm) were placed in the wells. An empty transwell without samples was served as the negative control group. The culture medium was changed every 3 days. After culturing for 3, 5, and 7 days respectively, the cell viability of each group was assessed using MTT assay (Chen Y. Z. et al., 2013). The optical density (OD) was measured at a wavelength of 492 nm using a microplate reader (Multiskan MK3, Thermo Labsystems Co., Shanghai, PRC). According to ISO 10993–5 (International Standards Organization, 2009), cell viability was calculated as a percentage using the following formula:

$$\text{Cell Viability}\%=\left({\text{OD}}_{\text{test}\;\text{material}}/{\text{OD}}_{\text{negative}\;\text{control}}\right)\times l00\%$$

The cell toxicity grades were defined as follows: 0 (cell viability ≥100%), 1 (75%–99%), 2 (50%–74%), 3 (25%–49%), 4 (1%–24%), and 5 (0%) (Lü et al., 2009; Chen et al., 2018).

### 2.3 Alkaline phosphatase activity assay

MG-63 cells were seeded at a density of 3 × 10^4^ cells per well in a 24-well plate and incubated overnight. The MEM medium was then replaced with osteogenic induction medium (100 nM dexamethasone, 10 mM β-glycerophosphate, and 50 μg/mL L-ascorbic acid (Sigma-Aldrich, St. Louis, MO, United States)). Transwells containing the test samples (diameter 5 mm × thickness 2 mm) were placed in the 24-well plate, and co-cultured with MG-63 cells for 3, 5, and 7 days respectively. After removing the samples, transwells and culture medium, the cells were washed twice with PBS. Then, 500 μL of 10 mM Tris-HCl buffer (pH 7.6) containing 0.1% Triton X-100 was added to each well. The ALP activity and total protein concentration of the cells were measured according to the instructions of the ALP assay kit (Nanjing Jiancheng Chemical Industrial Co., Ltd., Nanjing, China) (Chen et al., 2018). ALP activity was reported as nanomoles of p-nitrophenol produced per microgram of total protein, and the percentage of the sample group was calculated relative to the negative control group. An empty transwell was served as the negative control group.

### 2.4 Calcium ion concentration measurement

Samples of the three materials (diameter 5 mm × thickness 2 mm) were placed in a 24-well plate with one sample per well. Blank wells without the test materials were used as the negative control group. Each well was filled with 1 mL of MEM medium, which was changed every 3 days. After culturing for 3, 5, and 7 days respectively in a humidified atmosphere containing 5% CO_2_ at 37°C, 100 μL of the culture medium was collected and the calcium ion concentration was measured using an AU5800 fully automated biochemical analyzer (Beckman Coulter Inc., Brea CA, United States) (Liu H. et al., 2020).

### 2.5 Gene expression profiling microarray experiment

MG-63 cells were seeded at a density of 7 × 10^4^ cells/mL in a 6-well plate with 3 mL per well. After 24 h, the MEM medium was replaced with osteogenic induction medium. Three types of test samples (diameter 20 mm × thickness 1 mm) in transwells were co-cultured with MG-63 cells for 5 days, with an empty transwell without samples serving as the negative control group. Cells were rinsed with PBS (pH 7.2) twice, and total RNA from each group was extracted using Trizol reagent (Invitrogen Corporation, Grand Island, NY, United States). The quantity and quality of RNA were measured using a NanoDrop ND-1000 spectrophotometer (Thermo Fisher Scientific, Waltham, MA, United States), and the RNA integrity was assessed by standard denaturing agarose gel electrophoresis. The gene expression profiles of the cells in each sample were analyzed using a whole-genome oligo microarray (4 × 44K; Agilent Technologies, Inc., Santa Clara, CA, United States) (performed by Shanghai Kangcheng Biotechnology Company). Genes with a fold change ≥2 or ≤0.5 and a *p*-value <0.05, compared to the negative control group, were defined as upregulated or downregulated, respectively (Lü et al., 2009; Lü et al., 2010; Lü et al., 2014).

### 2.6 Bioinformatics analysis

First, the differential expression genes were analyzed using the DAVID online database (https://david.ncifcrf.gov/, 6 April 2023 version) to perform Gene Ontology (GO) analysis. Then, genes related to bone and tooth development were selected from the functional categories, and further analysis was conducted on the Kyoto Encyclopedia of Genes and Genomes (KEGG) pathways in which these genes are involved. And pathways at least two differentially expressed genes involved were selected.

### 2.7 Real-time fluorescent quantitative PCR analysis (qRT-PCR)

Four differentially expressed genes (*ENPP*1, *NPY*, *NOG* and *P*2*RX*7) associated with osteogenic/odontogenic differentiation of MG-63 cells were select for qRT-PCR analysis. *GAPDH* was served as the internal reference, and the primer sequences were listed in Table 1. The experiment was conducted in Shanghai Kangcheng Biotechnology Company.

**TABLE 1:** Sequences of the used primers for the RT-PCR experiment.

Gene	Forward primer	Reverse primer
*ENPP*1	5′-CCC​TTT​GGA​CAT​CCT​ATA​CCG​T-3′	5′-CTT​TCT​TCA​GCA​TAC​TTT​CGC​AG-3′
*NPY*	5′-CGA​CAC​TAC​ATC​AAC​CTC​ATC​A-3′	5′-GGT​CTT​CAA​GCC​GAG​TTC​TG-3′
*NOG*	5′-ACC​GCC​TCC​AAC​CAG​TT-3′	5′-GCA​ACA​ACC​AGA​ATA​AGT​CTC​T-3′
*P*2*RX*7	5′-GCC​CTG​TGT​GGT​CAA​CGA​AT-3′	5′-AGG​AAT​CGG​GGG​TGT​GTC​A-3′
*GAPDH*	5′-GGG​AAA​CTG​TGG​CGT​GAT-3′	5′-GAG​TGG​GTG​TCG​CTG​TTG​A-3′

### 2.8 Statistical analysis

All data were presented as mean ± standard deviation (mean ± SD). Statistical analysis of the data was performed using one-way analysis of variance (ANOVA), followed by Fisher’s least significant difference (LSD) test or Dunnett’s test for multiple comparisons using SPSS software (version 11.50; SPSS Inc., Chicago, IL, United States). Differences with *p* < 0.05 were considered statistically significant.

## 3 Results

### 3.1 Cell viability

The cell viability at different time points following the treatment with the three materials is shown in Table 2. Except for day 5 and day 7 for MTA, and day 5 for GIC (*p* < 0.05), there were no statistically significant differences in cell viability between the test groups and the negative control group (*p* > 0.05). The toxicity level of the experimental materials was classified as grade 1.

**TABLE 2:** Cell viability and cytotoxicity grade for test materials in MTT assay (n = 4).

Test groups	3 days		5 days		7 days	
	Cell viability (%)	Toxicity grade	Cell viability (%)	Toxicity grade	Cell viability (%)	Toxicity grade
Negative control	100.00 ± 3.39	0	100.00 ± 6.70	0	100.00 ± 6.28	0
NRFM-Zr	98.40 ± 11.77	1	93.26 ± 9.25	1	95.80 ± 12.18	1
MTA	93.40 ± 9.42	1	80.91 ± 10.27^a^	1	84.94 ± 3.75^a^	1
GIC	86.48 ± 8.84	1	81.86 ± 8.30^a^	1	91.99 ± 3.99	1

The superscript letters indicate statistical signifificance levels compared with the negative control at the same time (one-way ANOVA, followed by Dunnett’s test. ^a^ *p* < 0.05; ^b^ *p* < 0.01).

### 3.2 Alkaline phosphatase activity

**FIGURE 1:**
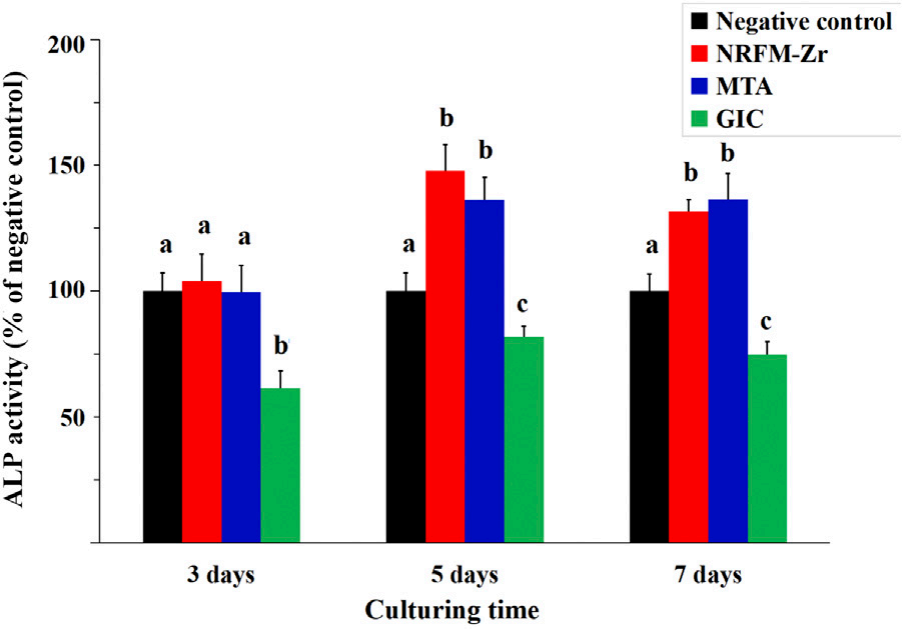
ALP activity of NRFM-Zr, MTA, GIC and negative control at various time intervals. Different letters above the bars indicate significant differences between the materials at the same time (one-way ANOVA followed by Fisher’s LSD test, *p* < 0.05).

Figure 1 shows that the ALP activity in the NRFM-Zr and MTA groups was significantly higher than the negative control on day 5 and day 7 (*p* < 0.05), while there was no significant difference on day 3 (*p* > 0.05). The ALP activity in the GIC group was significantly lower than the other three groups at all intervals (*p* < 0.05).

### 3.3 Calcium ion concentration

The calcium ion concentration of the test materials is shown in Table 3. Among them, the calcium ion concentration NRFM-Zr was significantly higher than the other three groups at all intervals (*p* < 0.05), while that of GIC was lower than the other groups (*p* < 0.05). Compared to the negative control group, MTA showed a higher calcium ion concentration on day 3 (*p* < 0.05), while there were no significant differences on day 5 and day 7 (*p* > 0.05).

**TABLE 3:** Calcium ion concentration of test materials (n = 4).

Test groups	3 days (mmol/L)	5 days (mmol/L)	7 days (mmol/L)
Negative control	1.19 ± 0.03^a^	1.17 ± 0.03^a^	1.18 ± 0.02^a^
NRFM-Zr	4.85 ± 0.23^b^	1.60 ± 0.29^b^	1.45 ± 0.15^b^
MTA	1.45 ± 0.04^c^	1.20 ± 0.03^a^	1.19 ± 0.12^a^
GIC	0.65 ± 0.16^d^	0.90 ± 0.09^c^	1.01 ± 0.03^c^

Different letters indicate significant differences between the materials at the same time (one-way ANOVA, followed by Fisher’s LSD test, *p* < 0.05).

### 3.4 Gene expression profiling microarray experiment

Table 4 shows the number of differentially expressed genes (fold change ≥2 or ≤0.5, *p* < 0.05) in the NRFM-Zr and MTA groups, which were 2915 and 2254, respectively, higher than the number in the GIC group (392). Additionally, in each group, the number of downregulated genes was greater than the number of upregulated genes. Detailed gene information can be found in Supplementary Tables S1–S3.

**TABLE 4:** The numbers of differentially expressed genes and their involved GO terms.

Test groups	Differentially expressed genes			GO terms		
	Upregulated	Downregulated	Total	Biological process	Cellular component	Molecular function
NRFM-Zr	1096	1819	2915	1715	189	192
MTA	955	1299	2254	1396	197	154
GIC	193	199	392	405	75	45

### 3.5 Bioinformatics analysis

#### 3.5.1 Gene ontology functional analysis

The total number of GO functional categories involved in the differentially expressed genes in the NRFM-Zr, MTA, and GIC groups were listed in Table 4. In order to analyze the effects of these three materials on osteogenesis and odontogenesis, the functional categories related to osteogenic/odontogenic differentiation were further screened from the biological process (BP) categories. The results were presented in Table 5.

**TABLE 5:** Osteogenesis-related and odontogenesis-related GO terms in NRFM-Zr and MTA treated groups.

GO BP terms		NRFM-Zr		MTA	
		Count	*p*-Value	Count	*p*-Value
Osteogenesis-related	GO:0030278∼Regulation of ossification	48	2.21E-05	35	2.19E-03
	GO:0001503∼Ossification	74	9.17E-05	57	1.92E-03
	GO:0030279∼Negative regulation of ossification	20	4.45E-03	17	5.98E-03
	GO:0045667∼Regulation of osteoblast differentiation	28	5.44E-03	21	3.57E-02
	GO:0045668∼Negative regulation of osteoblast differentiation	15	5.76E-03	13	6.61E-03
	GO:0045778∼Positive regulation of ossification	21	1.19E-02	17	2.52E-02
	GO:0001649∼Osteoblast differentiation	39	1.41E-02		
	GO:0002076∼Osteoblast development	6	4.47E-02		
Odontogenesis-related	GO:0042476∼Odontogenesis	30	2.30E-04	21	1.29E-02
	GO:0042475∼Odontogenesis of dentin-containing tooth	16	4.48E-02		

In the NRFM-Zr group, 8 GO functional categories related to osteogenesis and 2 categories related to odontogenesis were identified, involving 92 differentially expressed genes (43 upregulated and 49 downregulated; detailed information could be found in Supplementary Table S4). In the MTA group, 6 GO functional categories related to osteogenesis and 1 category related to odontogenesis were found, involving 71 differentially expressed genes (37 upregulated and 34 downregulated; detailed information can be found in Supplementary Table S5). There were 52 common genes between the two groups. However, the GIC group did not show any GO functional categories related to osteogenesis or odontogenesis.

#### 3.5.2 Kyoto encyclopedia of genes and genomes pathway analysis

**FIGURE 2:**
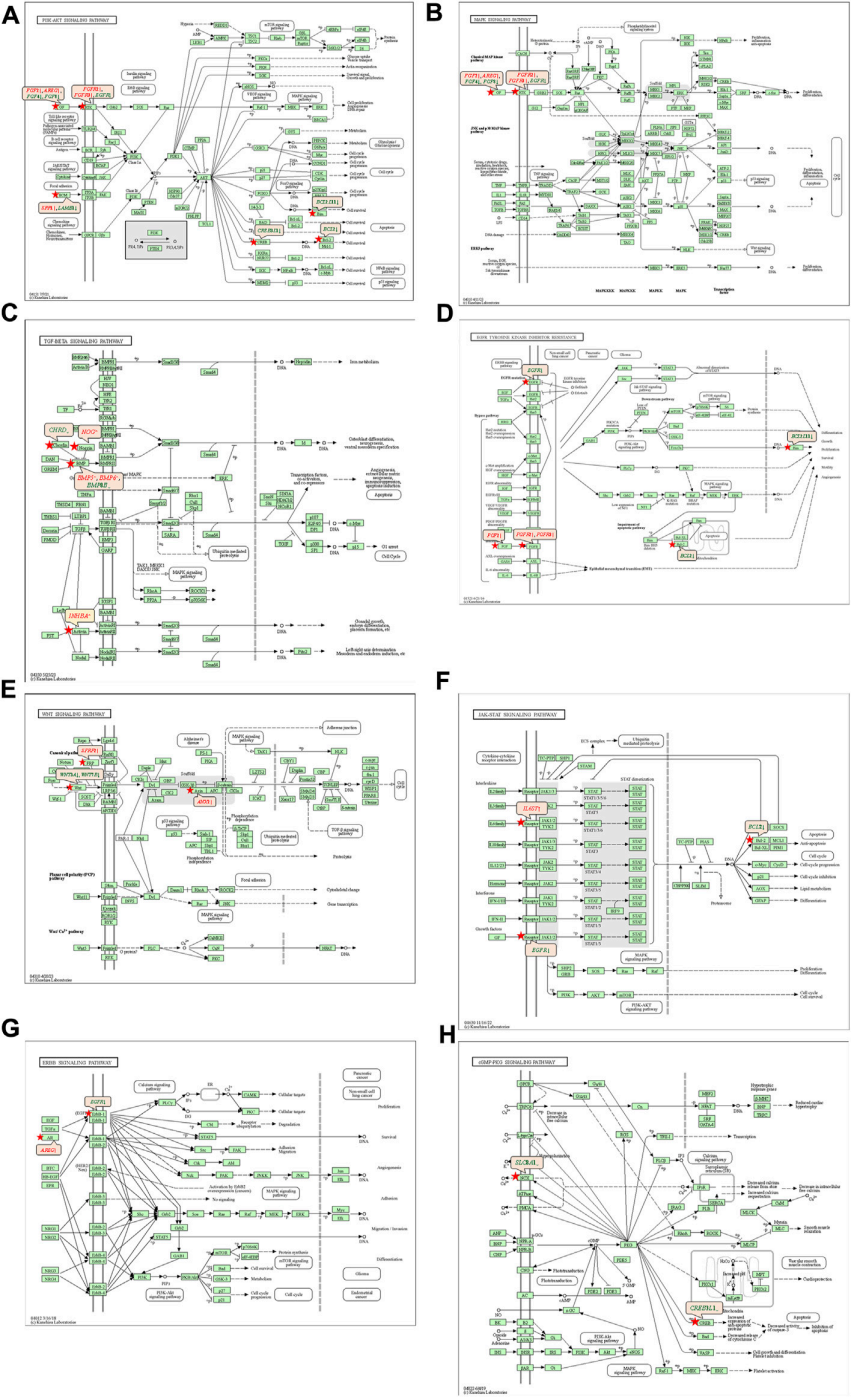
Pathways associated with osteogenic/odontogenic differentiation and the differentially epressed genes in the NRFM-Zr Group. **(A)** PI3K-Akt signaling pathway. **(B)** MAPK signaling pathway. **(C)** TGF-β signaling pathway. **(D)** EGFR tyrosine kinase inhibitor resistance. **(E)** Wnt signaling pathway. **(F)** JAK-STAT signaling pathway. **(G)** ErbB signaling pathway. **(H)** cGMP-PKG signaling pathway. Red letters indicate upregulated genes and green letters indicate downregulated genes.

**FIGURE 3:**
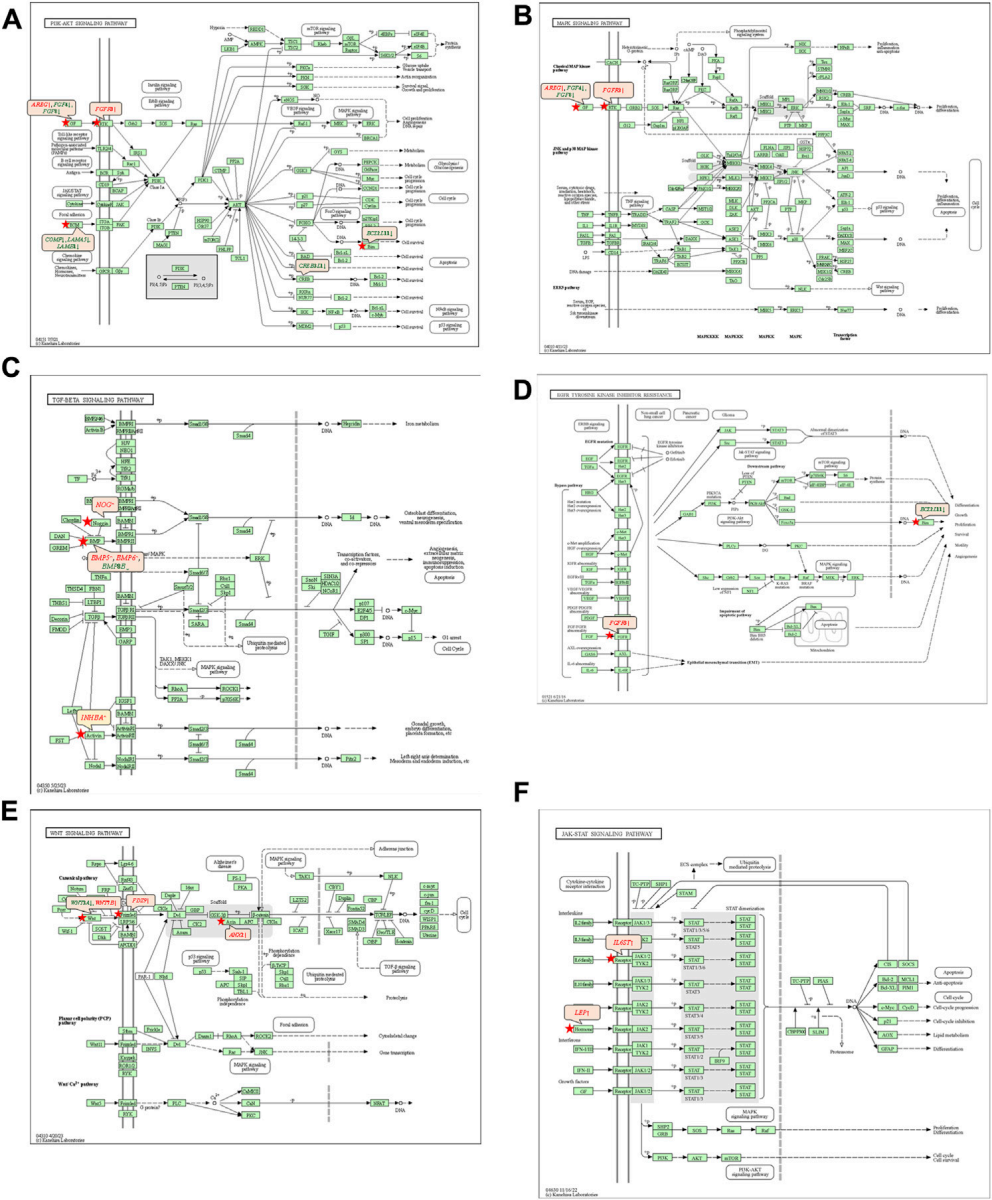
Pathways associated with osteogenic/odontogenic differentiation and the differentially expressed genes in the MTA Group. **(A)** PI3K-Akt signaling pathway. **(B)** MAPK signaling pathway. **(C)** TGF-β signaling pathway. **(D)** EGFR tyrosine kinase inhibitor resistance. **(E)** Wnt signaling pathway. **(F)** ErbB signaling pathway. Red letters indicate upregulated genes and green letters indicate downregulated genes.

The 92 genes identified from the NRFM-Zr group and the 71 genes from the MTA group, which were related to osteogenesis and odontogenesis as selected in Section 3.5.1, were subjected to KEGG pathway analysis. It was found that the differentially expressed genes in the NRFM-Zr group were involved in 89 pathways, while those in the MTA group were involved in 46 pathways. Among them, there were 8 pathways related to osteogenic/odontogenic differentiation in the NRFM-Zr group and 6 pathways in the MTA group. The detailed information could be found in Table 6. The pathway maps and the genes involved were shown in Figures 2, 3.

**TABLE 6:** Pathways and genes associated with osteogenic/odontogenic differentiation in the NRFM-Zr and MTA Groups.

No.	Pathway	NRFM-Zr	MTA
1	PI3K-Akt signaling pathway	12 ( AREG , BCL2L11 , FGF4 , FGF8 , FGFR3 , LAMB1 , BCL2 , CREB3L1 , EGFR , FGF2 , FGFR2 , SPP1 )	8 ( AREG , BCL2L11 , FGF4 , FGF8 , FGFR3 , LAMB1 , COMP , LAMA5 )
2	MAPK signaling pathway	7 ( AREG , FGF4 , FGF8 , FGFR3 , EGFR , FGF2 , FGFR2 )	4 ( AREG , FGF4 , FGF8 , FGFR3 )
3	TGF-β signaling pathway	6 ( BMP5 , BMP6 , BMP8B , INHBA , NOG , CHRD )	5 ( BMP5 , BMP6 , BMP8B , INHBA , NOG )
4	EGFR tyrosine kinase inhibitor resistance	6 ( BCL2L11 , FGFR3 , BCL2 , FGF2 , EGFR , FGFR2 )	2 ( BCL2L11 , FGFR3 )
5	Wnt signaling pathway	4 ( AXIN2 , WNT3A , WNT7B , SFRP2 )	4 ( AXIN2 , WNT3A , WNT7B , FZD9 )
6	JAK-STAT signaling pathway	3 ( IL6ST , BCL2 , EGFR )	2 ( IL6ST , LEP )
7	ErbB signaling pathway	2 ( AREG , EGFR )	
8	cGMP-PKG signaling pathway	2 ( CREB3L1 , SLC8A1 )	

Red letters indicate upregulated genes and green letters indicate downregulated genes.

### 3.6 qRT-PCR results

**FIGURE 4:**
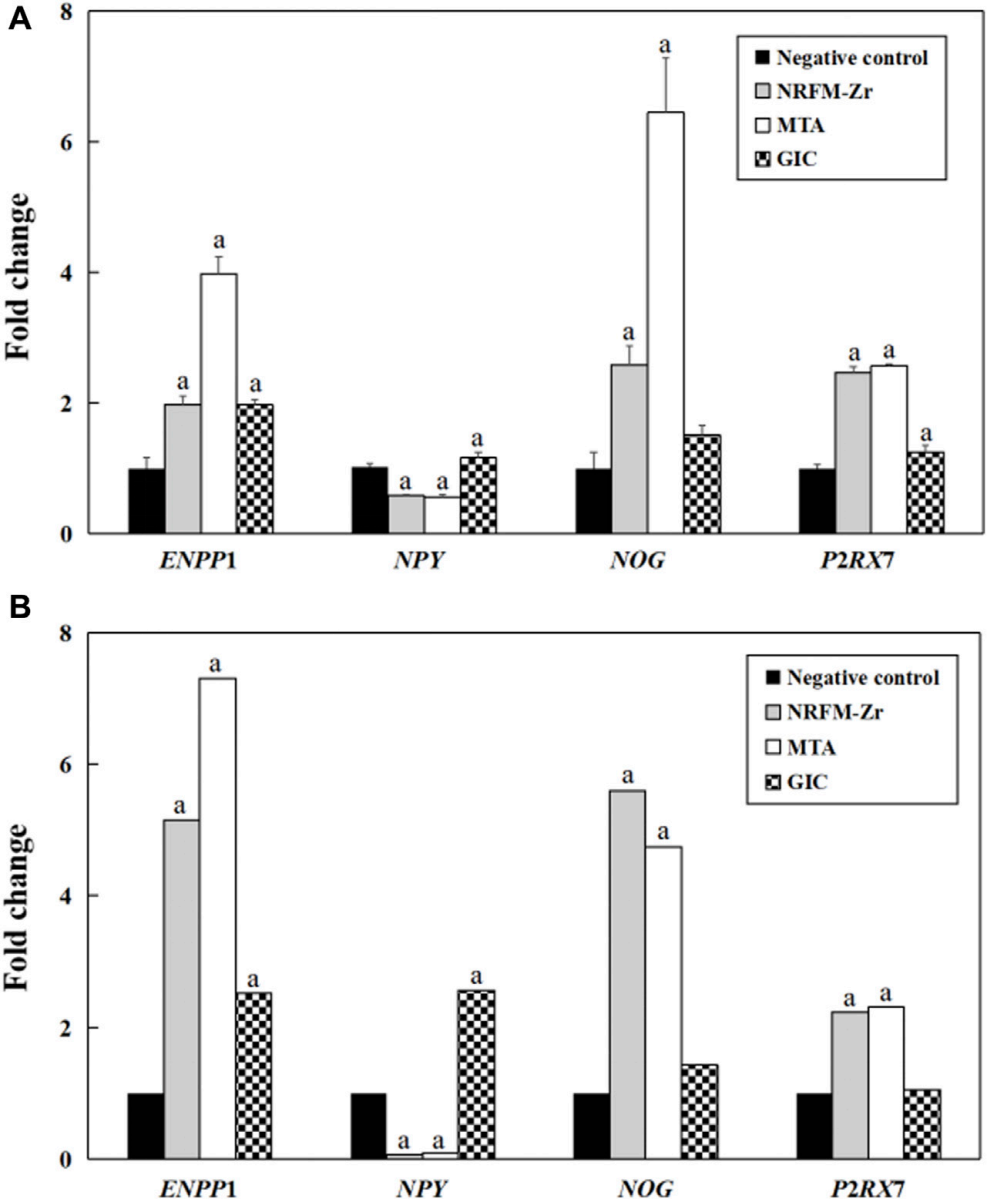
Validation of gene expression profiling microarray results by analyzing the expression levels of four genes with qRT-PCR. **(A)** qRT-PCR. **(B)** Gene expression profiling microarray. “a” represents *p* < 0.05 comparing with negative control.

Figure 4 shows the fold changes of the 4 genes (*ENPP*1, *NPY*, *NOG* and *P*2*RX*7) associated with osteogenesis and odontogenesis from qRT-PCR and gene expression profiling microarray. The expression trends of the two methods were largely consistent, demonstrating the reliability of the gene expression profiling microarray experiment results.

## 4 Discussion

### 4.1 Cell viability

Given that RFMs come into direct contact with periapical tissues, ideal RFMs should present desirable cytocompatibility. The results of this study (Table 2) showed that the cell viability of NRFM-Zr co-cultured with MG-63 cells ranged from 93.26% to 98.40% at three time points. Our previous research demonstrated that the extract of freshly prepared or set NRFM-Zr exhibited good cell compatibility with L929 mouse fibroblasts (Chen et al., 2018). These results indicate that NRFM-Zr has favorable cell compatibility. Additionally, this study also showed that the cell viability of MTA (at 5 and 7 days) and GIC (at 5 days) was significantly lower than the negative control group (*p* < 0.05), although their cell toxicity levels were also classified as grade 1. These findings are consistent with other studies (Keiser et al., 2000; Chen Y. Z. et al., 2013; Chen et al., 2018), such as the study by Keiser et al. (2000) which demonstrated that high concentrations of MTA extract had an inhibitory effect on human periodontal ligament cell proliferation. However, other studies have shown that MTA does not exhibit cytotoxicity towards human periodontal ligament cells (Gorduysus et al., 2007).

### 4.2 Alkaline phosphatase activity

Apart from desirable cytocompatibility, osteogenic/odontogenic performance is another important property of RFMs. ALP activity is a marker of early differentiation of osteoblasts (Fan et al., 2015), therefore, in this study, ALP activity was used to investigate the osteogenic/odontogenic differentiation potential of NRFM-Zr. The results showed that the NRFM-Zr group at day 5 and day 7 exhibited significantly higher ALP activity compared to the negative control group (*p* < 0.05). Our previous research also demonstrated that the extract of NRFM-Zr significantly increased the ALP activity of Saos-2 cells (Chen et al., 2018). Similar to NRFM-Zr, MTA at day 5 and day 7 significantly increased the ALP activity of MG-63 cells (*p* < 0.05), which is consistent with previous studies (Chang et al., 2014). For example, Chang et al. (2014) showed that MTA increased ALP activity and promoted mineralized nodule formation in human dental pulp cells. In contrast, the ALP activity of GIC was significantly lower than the negative control at all time points. Chen et al. (2002) found that GIC and resin-modified light-cured GIC inhibited the ALP activity of human dental pulp fibroblasts. Therefore, NRFM-Zr and MTA can promote the osteogenic/odontogenic differentiation of MG-63 cells, while GIC inhibits the osteogenic/odontogenic differentiation of MG-63 cells.

### 4.3 Calcium ion concentration

Calcium ions may play an important role in the formation and mineralization of hard tissues, therefore, the calcium ion concentration of the materials was also tested in this study. The results showed that the calcium ion concentration of NRFM-Zr ranged from 1.45 to 4.85 mmol/L, which was significantly higher than the negative control group. The increase of extracellular calcium ion concentration could cause a dramatic increase of intracellular calcium ion concentration through calcium channels (Zayzafoon, 2006). Jung et al. (2010) further showed that HA-released Ca^2+^ improved the MC3T3-E1 cell differentiation by increasing bone sialoprotein and osteopontin expression through L-type calcium channel which triggered calcium/calmodulin mediated calcium/calmodulin-dependent protein kinase 2 (CaM–CaMK2) pathway. Moreover, Maeno et al. (2005) demonstrated that slightly higher concentrations of calcium ions are beneficial for osteoblast differentiation and matrix mineralization. Therefore, the calcium ions released from the main components of NRFM-Zr, hydroxyapatite and carboxylate calcium salts, are believed to promote the osteogenic/odontogenic differentiation of MG-63 cells. Similar to NRFM-Zr, MTA exhibited a higher calcium ion concentration (1.45 mmol/L) at day 3 (*p* < 0.05). Many studies have indicated that MTA’s promotion of osteoblast differentiation is not only associated with higher calcium ion concentration but also with alkaline pH values (Bonson et al., 2004; Matsumoto et al., 2013; Gandolfi et al., 2014). In contrast to NRFM-Zr and MTA, GIC exhibited an acidic pH and lower calcium ion concentration (0.65–1.01 mmol/L), thereby exerting an inhibitory effect on the osteogenic/odontogenic differentiation of MG-63 cells.

### 4.4 Gene expression profiling microarray experiment and bioinformatics analysis

To further investigate the molecular mechanisms underlying NRFM-Zr’s promotion of osteogenic/odontogenic differentiation in MG-63 cells, this study conducted comprehensive analysis using gene expression profiling microarray and bioinformatics analysis. The results of the gene expression profiling microarray showed that the number of differentially expressed genes in the NRFM-Zr (2915) and MTA (2254) groups was much higher than in the GIC group (392), indicating that the former two had a greater impact on the gene expression profile of MG-63 cells than the latter. The results of GO functional analysis on the differentially expressed genes revealed that the 8 osteogenesis and 2 odontogenesis related categories involved in the NRFM-Zr group fully encompassed the 6 osteogenesis and 1 odontogenesis related categories in the MTA group (*p* < 0.05). Moreover, the two groups shared 52 consistently differentially expressed genes. These results suggested that NRFM-Zr and MTA might have some similar molecular mechanisms, with NRFM-Zr exerting a broader impact. In contrast, unlike the two aforementioned materials, the GIC group did not show any osteogenesis and odontogenesis related functional categories, which supported its lack of significant promotion on osteogenic/odontogenic differentiation in MG-63 cells at the molecular level. Therefore, further discussion on GIC was not conducted in the subsequent sections.

The KEGG pathway analysis revealed that the NRFM-Zr group had more pathways (8) and a greater number of genes (24) associated with osteogenic/odontogenic differentiation compared to the MTA group (6 pathways, 19 genes). Among these pathways, 6 were common to both groups, while the NRFM-Zr group had 2 unique pathways (Table 6). This indicated that NRFM-Zr had a greater impact on the osteogenic/odontogenic differentiation of MG-63 cells compared to MTA.

#### 4.4.1 PI3K-AKT signaling pathway and MAPK signaling pathway

From Table 6 and Figure 2, and Figure 3, it could be observed that genes involved in the PI3K-AKT signaling pathway in the NRFM-Zr/MTA group included all genes involved in the MAPK signaling pathway, EGFR tyrosine kinase inhibitor resistance pathway, and ErbB signaling pathway. Therefore, these four pathways would be discussed together.

The PI3K-Akt signaling pathway consists of PI3K and its downstream molecule Akt, which has been shown to be crucial for osteogenic differentiation, growth, and survival (Liu Y. et al., 2020). NRFM-Zr activates the PI3K-Akt signaling pathway in MG-63 cells, resulting in the downregulation of downstream genes *CREB*3*L*1, *BCL*2, and *BCL*2*L*11. *BCL*2 is located downstream of *CREB*3*L*1, and studies by Moriishi et al. (2011, 2014) had shown that low expression of *BCL*2 promoted osteogenic differentiation, while overexpression of *BCL*2 inhibited osteogenic differentiation, reduced the progression of osteoblasts, and led to osteoblast apoptosis. Therefore, the downregulation of *BCL*2 in this study might promote osteogenic differentiation of MG-63 cells. In addition, a study by Chen Y. T. et al. (2013) demonstrated that inhibition of Bim protein (BCL2L11) expression protected osteoblasts from apoptosis. Liang et al. (2008) also showed that partial protection against cell apoptosis could be achieved by specifically knocking out *Bim* in osteoblasts. In this study, the downregulation of the *BCL*2*L*11 gene might protect MG-63 cells from apoptosis. Although MTA also activated the PI3K-Akt signaling pathway in MG-63 cells, it only exhibited downregulation of the *BCL*2*L*11 gene downstream, suggesting that it might only play a role in inhibiting apoptosis, and its osteogenic differentiation potential might be lower than that of NRFM-Zr.

**FIGURE 5:**
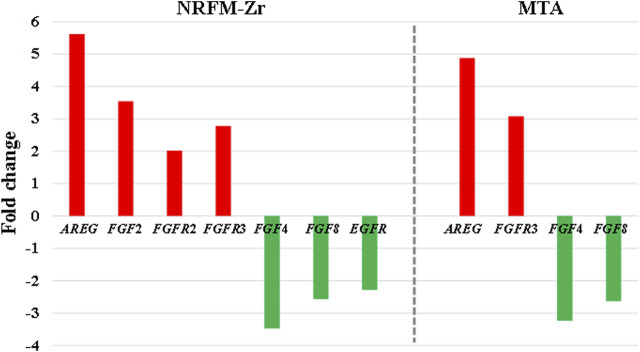
The fold changes of differentially expressed genes involved in the MAPK signaling pathway in the NRFM-Zr and MTA groups. To facilitate comparison, the fold changes of downregulated genes were represented as the negative reciprocal of their expression values.

The MAPK signaling pathway mediates various physiological processes, including cell growth, development, proliferation, differentiation, aging, and death. Studies have shown that the MAPK signaling pathway promotes osteoblast proliferation, differentiation, and delayed apoptosis through various pathways (Luo et al., 2005; Greenblatt et al., 2022). In the NRFM-Zr group, there were 7 genes involved in this pathway, while the MTA group had 4 genes (Table 6). *AREG* is associated with bone physiology and intermittent parathyroid hormone treatment-mediated bone anabolic metabolism, and overexpression of *AREG* in osteoblasts could induce a high bone mass phenotype (Vaidya et al., 2015). *FGF*2, *FGF*4, and *FGF*8 belong to the fibroblast growth factor family. *FGF*2 not only directly participated in the regulation of osteogenic differentiation but also synergized with Wnt/beta-catenin, bone morphogenetic protein, and MAPK signaling pathways to regulate bone metabolism (Ahn et al., 2009). *FGF*4 could affect osteogenic differentiation through the activation of MAPK-mediated signaling pathways (Kook et al., 2013). *FGF*8 was an effective stimulator of Wnt/β-catenin protein activity and an important factor in tooth development (Kimura et al., 2022). Enhanced functionality of *FGFR*2 could enhance Erk1/2 phosphorylation in bone marrow mesenchymal stem cells (BMSCs), thereby promoting osteogenic differentiation (Zhang et al., 2022). Downregulation of FGFR3 inhibited osteogenic differentiation of BMSCs in *TBXT* gene mutant mice (Su et al., 2023). EGFR is a tyrosine kinase involved in regulating cell homeostasis. EGFR and its ligands regulate key biological processes in cells, such as proliferation, survival, differentiation, and migration. Downregulation of *EGFR* had an inhibitory effect on osteogenesis (Ge et al., 2021). Among the 7 differentially expressed genes in the NRFM-Zr group, 4 genes (upregulated *AREG*, *FGF*2, *FGFR*2, *FGFR*3) promoted osteogenic differentiation, while 3 genes (downregulated *FGF*4, *FGF*8, *EGFR*) hindered osteogenic differentiation. In the MTA group, there were 2 genes each that promoted (upregulated *AREG*, *FGFR*3) or hindered (downregulated *FGF*4, *FGF*8) osteogenic differentiation. Overall, NRFM-Zr (the sum of fold change of was +5.64) exhibited a greater osteoinductive effect than MTA (the sum of fold change was + 2.09) (Figure 5). The EGFR signaling pathway (EGFR tyrosine kinase inhibitor resistance pathway in this study), is an important regulatory pathway in bone regulation and plays a crucial anabolic role in bone metabolism (Zhu et al., 2011). Among the 6 differentially expressed genes involved in this pathway induced by NRFM-Zr, 4 genes (upregulated *FGF*2, *FGFR*2, *FGFR*3, and downregulated *BCL*2) were favorable for osteoblastic differentiation, while only 1 gene (downregulated *EGFR*) was unfavorable. In contrast, the MTA group had only 1 gene (upregulated *FGFR*3) that was favorable for osteoblastic differentiation. This suggested that NRFM-Zr might have a greater potential for osteoinduction compared to MTA in terms of its effect on the EGFR signaling pathway.

The ErbB family couples extracellular growth factor ligands to intracellular signaling pathways that regulate various biological responses, including proliferation, differentiation, cell motility, and survival. This pathway was specific to the NRFM-Zr group and involved the upregulation of *AREG* and downregulation of *EGFR*. The fold change of *AREG* was greater than that of *EGFR* (as shown in Figure 5), indicating an overall promotion of osteoinductive effects.

#### 4.4.2 TGF-β signaling pathway

The TGF-β signaling pathway plays a crucial role in various cellular functions, including proliferation, apoptosis, differentiation, and migration. During osteogenic differentiation, the TGF-β signaling pathway transmits signals through type I and type II serine/threonine kinase receptors (Almuraikhi, 2023).

**FIGURE 6:**
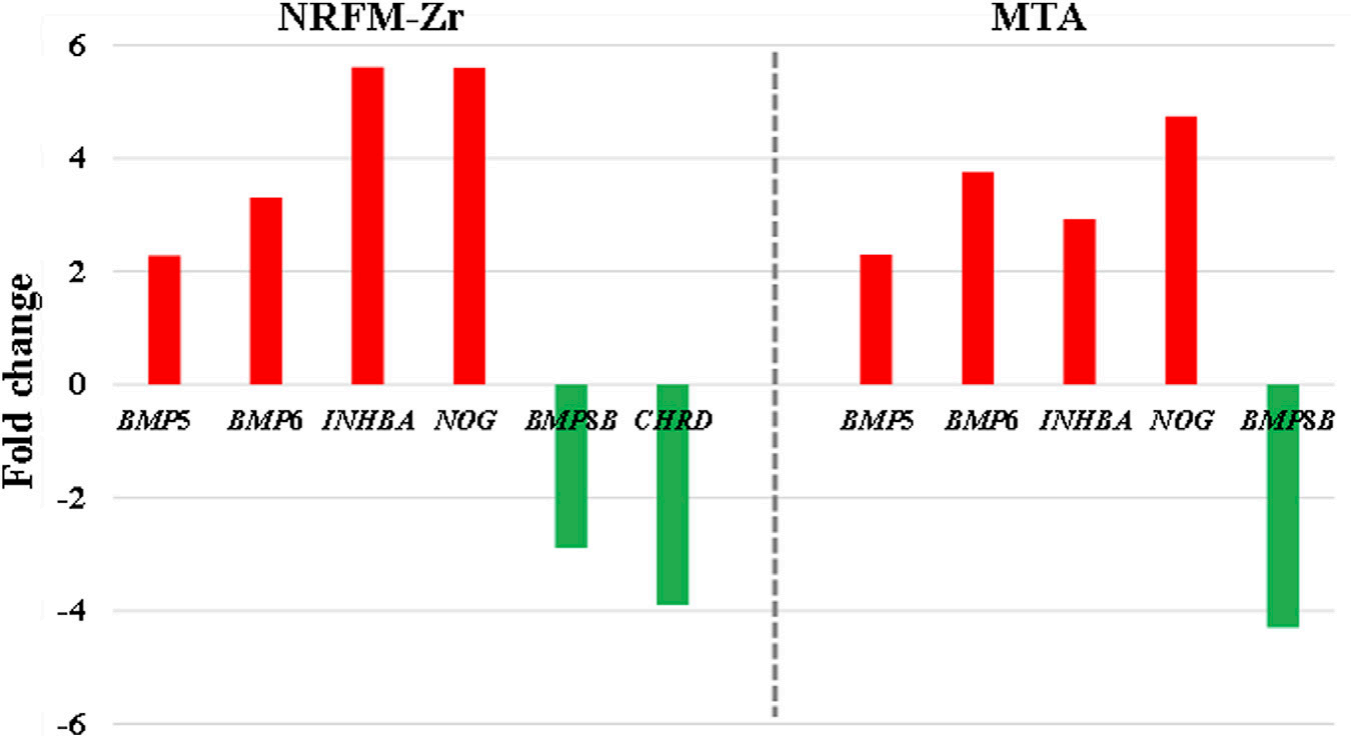
The fold changes of differentially expressed genes involved in the TGF-β signaling pathway between the NRFM-Zr and MTA groups. To facilitate comparison, the fold changes of downregulated genes were represented by the negative reciprocal of their expression values.

In the NRFM-Zr group, there were 6 differentially expressed genes involved in this pathway. Among them, 4 genes (*BMP*5, *BMP*6, *INHBA*, and *NOG*) were beneficial for osteogenic differentiation (Hopwood et al., 2007; Zou et al., 2013), while 2 genes (*BMP*8*B* and *CHRD*) were unfavorable for osteogenic differentiation (Macsai et al., 2012; Wang et al., 2022). The MTA group also contained the same 4 beneficial genes and 1 unfavorable gene (*BMP*8*B*) for osteogenic differentiation. However, overall, NRFM-Zr (the sum of fold change was +10.02) might have a greater osteoinductive effect than MTA (the sum of fold change was + 9.42) (Figure 6).

#### 4.4.3 Wnt signaling pathway

Wnt proteins are essential for the fundamental developmental processes in various species and organs, including cell fate specification, progenitor cell proliferation, and control of asymmetric cell division. Upon activation of the Wnt signaling pathway, β-catenin translocates to the nucleus and becomes stabilized, where it then binds to DNA-binding proteins of the TCF/LEF family and regulates osteogenesis through target genes mediated by Wnt (Sharma et al., 2022).

Both NRFM-Zr and MTA upregulate the downstream gene *AXIN*2 in the Wnt signaling pathway. *AXIN*2 facilitates the binding activity of I-SMAD and ubiquitin protein ligase, and it is involved in the regulation of centromere sister chromatid cohesion. It plays a role in multiple processes, including negative regulation of osteoblast differentiation and regulation of chondrocyte development. Previous study found that IL-35 enhanced the expression of key components, such as p-catenin and *Axin*2, in the Wnt/β-catenin pathway during the differentiation of mesenchymal stem cells into osteoblasts. This indicated that the upregulation of *Axin*2 suggested the occurrence of osteogenic differentiation (Li et al., 2023). The fold change of *AXIN*2 in the NRFM-Zr group (2.21) was slightly higher than that in the MTA group (2.02), suggesting that NRFM-Zr might have a slightly superior effect on osteogenic differentiation compared to MTA.

#### 4.4.4 JAK-STAT signaling pathway

In mammals, the JAK/STAT pathway is a major signaling conduction mechanism for various cytokines and growth factors. The JAK/STAT pathway is involved in bone homeostasis and plays an important role in the proliferation and differentiation of certain cell types, including osteoblasts and osteoclasts (Sanpaolo et al., 2020). Compared to MTA, NRFM-Zr inhibited the expression of the downstream gene *BCL*2 in this pathway. The downregulation of BCL2 promoted osteoblast differentiation.

#### 4.4.5 cGMP-PKG signaling pathway

cGMP is an intracellular second messenger that mediates the actions of nitric oxide and natriuretic peptides, regulating a wide range of physiological processes. Elevated intracellular cGMP levels exert their physiological effects through cGMP-dependent protein kinase (PKG), cGMP-regulated phosphodiesterases, and cGMP-gated cation channels, with PKG being a major mediator. PKG can also open mitochondrial ATP-sensitive K^+^ channels, leading to the release of ROS for protective effects. This pathway is specific to the NRFM-Zr group and involved the downstream gene *CREB*3*L*1, and the downregulation of CREB3L1 was unfavorable for osteoblast differentiation (Hansamuit et al., 2020).

### 4.5 qRT-PCR

The comparison between the validation experiments by qRT-PCR and the gene expression profiling microarray results showed that although there were certain differences in the expression levels of genes such as *ENPP*1, the majority of the results were consistent. This indicated that the results obtained from the gene expression profiling microarray were reliable and reproducible.

## 5 Conclusion

In this study, gene expression profiling microarray and bioinformatics techniques were employed to comparatively investigate the impact molecular mechanisms underlying osteogenic/odontogenic differentiation of three different types of root-end filling materials (hydroxyapatite-based NRFM-Zr, calcium silicate-based MTA, and GIC) on MG-63 cells. The results revealed the following findings: 1) Hydroxyapatite-based and calcium silicate-based root-end filling materials shared similar molecular mechanisms in influencing osteogenic/odontogenic differentiation. 2) NRFM-Zr may promote the osteogenic/odontogenic differentiation of MG-63 cells through eight signaling pathways including PI3K-Akt and MAPK, as well as affecting the expression of related genes in these pathways which promote differentiation and inhibit cell apoptosis. 3)The inducing efficacy of NRFM-Zr was found to be superior to MTA due to desirable cell viability, ALP activity and calcium ion concentration (Table 7).

**TABLE 7:** Comparison of NRFM-Zr, MTA and GIC.

Contents	NRFM-Zr	MTA	GIC
**Cell viability (**%**)**	93.26–98.40	80.91–93.40	81.86–91.99
**ALP activity**	Significantly increased on day 5 and day 7	Significantly increased on day 5 and day 7	Lower than ALP activity in NRFM-Zr and MTA group
**Ca** ^**2+**^ **concentration (mmol/L)**	1.45–4.85	1.19–1.45	0.65–1.01
**GO functional categories related to osteogenesis and odontogenesis**	10	7	0
**Genes related to osteogenesis and odontogenesis**	92	71	0
**Pathways related to osteogenesis and odontogenesis**	8	6	0
(1)PI3K-Akt signaling pathway	Downregulation of *BCL*2 promoted differentiation	Downregulation of *BCL*2*L*11 inhibited apoptosis	
	Downregulation of *BCL*2*L*11 inhibited apoptosis		
(2)MAPK signaling pathway	4 genes favored osteogenic differentiation, while 3 genes hindered osteogenic differentiation	2 genes promoted osteogenic differentiation and 2 genes hindered osteogenic differentiation	
	The sum of fold change was +5.64 (greater than 0, indicating a favorable effect on differentiation)	The sum of fold change was +2.09	
(3)EGFR tyrosine kinase inhibitor resistance	4 genes promoted osteogenic differentiation and only 1 gene hindered osteogenic differentiation	1 gene promoted osteogenic differentiation	
(4)ErbB signaling pathway	The expression levels of *AREG* was greater than *EGFR*.		
(5)TGF-β signaling pathway	4 genes promoted osteogenic differentiation and 2 genes inhibited osteogenic differentiation	4 genes promoted osteogenic differentiation and 1 gene inhibited osteogenic differentiation	
	The sum of fold change was +10.02	The sum of fold change was +9.42	
(6) Wnt signaling pathway	The fold change of *AXIN*2 was 2.21	The fold change of *AXIN*2 was 2.02	
(7)JAK-STAT signaling pathway	The downregulation of *BCL*2 promoted differentiation		
(8)cGMP-PKG signaling pathway	The downregulation of *CREB*3*L*1 inhibited apoptosis		

Based on the results of this study, not only have we found that NRFM-Zr appears as a suitable root-end filling material, but we have also basically elucidated its molecular mechanism in promoting osteogenic/odontogenic differentiation. However, further investigations are still needed to validate the key gene or pathway function obtained in this paper, and *in vivo* animal experiments need to be carried out to further verify its osteogenic/odontogenic properties and lay a foundation for future clinical applications.

## Data Availability

The original contributions presented in the study are included in the article/Supplementary Material, further inquiries can be directed to the corresponding author.

## Supplementary material

SUPPLEMENTARY TABLE S1 Differentially expressed genes of NRFM-Zr.

SUPPLEMENTARY TABLE S2 Differentially expressed genes of MTA.

SUPPLEMENTARY TABLE S3 Differentially expressed genes of GIC.

SUPPLEMENTARY TABLE S4 Osteogenesis-related and odontogenesis-related differentially expressed genes of NRFM-Zr.

SUPPLEMENTARY TABLE S5 Osteogenesis-related and odontogenesis-related differentially expressed genes of MTA.
